# Defeat Senate Bill 1552

**Published:** 1896-06

**Authors:** 


### DEFEAT SENATE BILL 1552.

Every true friend and advocate of scientific investigation and research which have added so much to the world's progress in the sphere of medicine and surgery through experimental work on the lower animals, and by which so much relief has been afforded the suffering ones of the human family, will with one accord join in the dignified and wise protest of the American Medical Association against Senate Bill No. 1552, which has for its aims prohibitory provisions against experiments on the lower animals. In these days of so much new light upon the treatment of diseases in the better and more reasonable lines of research, prohibitive measures of this kind are almost criminal; for none would sustain or pardon the testing of these agents and measures on the human family until their thorough, careful, and patient trial and study from every standpoint upon the lower animals, which this measure is designed to retard and prevent. This should receive the emphatic protest of every veterinarian in the land, and we trust our readers will take this opportunity of writing their representatives in the lower House, and thus restrain the adoption of a measure that is fraught with so much danger to the true progress of medical science.

